# Octopus-inspired adhesive skins for intelligent and rapidly switchable underwater adhesion

**DOI:** 10.1126/sciadv.abq1905

**Published:** 2022-07-13

**Authors:** Sean T. Frey, A. B. M. Tahidul Haque, Ravi Tutika, Elizabeth V. Krotz, Chanhong Lee, Cole B. Haverkamp, Eric J. Markvicka, Michael D. Bartlett

**Affiliations:** ^1^ Materials Science and Engineering, Iowa State University, Ames, IA 50010, USA.; ^2^ Mechanical Engineering, Soft Materials and Structures Lab, Virginia Tech, Blacksburg, VA 24061, USA.; ^3^ Macromolecules Innovation Institute, Virginia Tech, Blacksburg, VA 24061, USA.; ^4^ Department of Mechanical and Materials Engineering, Smart Materials and Robotics Laboratory, University of Nebraska-Lincoln, Lincoln, NE 68588, USA.; ^5^ Department of Electrical and Computer Engineering, University of Nebraska-Lincoln, Lincoln, NE 68588, USA.

## Abstract

The octopus couples controllable adhesives with intricately embedded sensing, processing, and control to manipulate underwater objects. Current synthetic adhesive–based manipulators are typically manually operated without sensing or control and can be slow to activate and release adhesion, which limits system-level manipulation. Here, we couple switchable, octopus-inspired adhesives with embedded sensing, processing, and control for robust underwater manipulation. Adhesion strength is switched over 450× from the ON to OFF state in <50 ms over many cycles with an actively controlled membrane. Systematic design of adhesive geometry enables adherence to nonideal surfaces with low preload and independent control of adhesive strength and adhesive toughness for strong and reliable attachment and easy release. Our bio-inspired nervous system detects objects and autonomously triggers the switchable adhesives. This is implemented into a wearable glove where an array of adhesives and sensors creates a biomimetic adhesive skin to manipulate diverse underwater objects.

## Teaser

Octopus-inspired switchable adhesives are integrated with sensing, processing, and control for robust underwater manipulation.

## INTRODUCTION

Strong and reversible attachment to underwater surfaces and objects is a substantial challenge (*1*, *2*). Unlike dry environments where adhesives can use van der Waals forces, electrostatic forces, and hydrogen bonds, wet or underwater surfaces markedly reduce the effectiveness of these mechanisms (*3*–*6*). Regardless, nature has numerous examples of organisms that have developed the ability to create strong attachment in moist or submerged environments. Mussels secrete specialized adhesive proteins and create an adhesive plaque to attach to moist surfaces (*7*, *8*), frogs channel fluid through structured toe pads to activate capillary and hydrodynamic forces (*9*–*14*), and cephalopods like the octopus use suckers to generate adhesion and suction forces (*15*–*23*). Cephalopod grippers are particularly attractive for underwater gripping as the adhesives are reversible, attachment can be activated quickly, and adhesion can be achieved on diverse substrates under dry and wet conditions (*24*–*26*). An additional sophistication in nature is a rich display of sensing and control that accompanies the adhesive system (*27*–*29*). The cephalopod sensing system consists of a photoreception vision system through their eyes; mechanoreceptors to detect fluid flow, pressure, and contact; and chemoreception tactile sensors (*30*, *31*). This capability provides information on attachment and proximity to objects, enabling the organism to display active gripping and releasing for efficient and reliable attachment (*32*). Moreover, an octopus can have more than 2000 suckers distributed across its eight arms, where each adhesive is independently controlled to activate or release adhesion (*33*, *34*). This combination of adhesion tunability, sensing, and control is unmatched in synthetic adhesives.

Switchable adhesives can generate strong adhesion yet be removed on demand with a prescribed trigger and then be reused (*35*). In these systems, a trigger, such as a mechanical, electromagnetic, fluidic, or thermal stimuli, results in a change in contact area, mechanical properties, or near-interface characteristics to modulate adhesion. In the active, pressure-driven systems observed in cephalopods, a pressure differential between the sucker chamber and the surrounding medium is created to generate a force used for attaching (*23*, *36*). The sucker is then actuated again for release. This mechanism functions in dry or unsubmerged environments, allowing for attachment and release in diverse environments. The characteristics and mechanisms of the cephalopod have inspired numerous mimics such as tentacle-like soft robotic grippers (*37*–*39*), passive and active microstructured surfaces (*16*, *18*, *40*, *41*), and active systems controlled by dielectric elastomer actuators and pneumatic pumps (*15*, *17*, *19*, *42*–*44*). However, cephalopods have a distinct advantage over synthetic adhesives as they have a prominent sensing system for proximity detection and mechanoreceptors to detect contact with surfaces, enabling active control of the adhesive elements (*32*). Sensing the proximity of a surface in synthetic systems that work in air and underwater can be achieved through a few different methodologies. This includes optical proximity sensors that use lasers (*45*–*47*) or camera-based vision systems (*47*, *48*), and sound-based range sensors (*49*). However, because of the size of these sensor systems, they are rarely integrated with synthetic adhesives, which limits manipulation or autonomous grasping capabilities in uncontrolled environments.

**Fig. 1.**
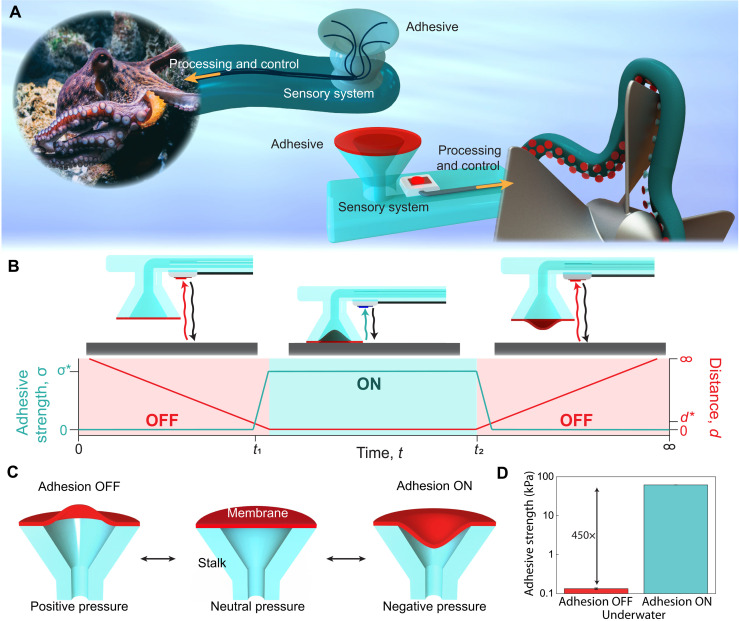
Octopus-inspired switchable, sensorized underwater adhesive. (**A**) Illustration of the octopus adhesive system and sensorized, octopus-inspired adhesive system, showing the adhesive and sensory system integrated with processing and control to sense objects and switch adhesion. (**B**) Schematic showing a synthetic adhesive with an integrated micro-LIDAR optical sensor where the adhesion goes from an OFF state to an ON state with an adhesive strength σ* once the sensor is triggered at a distance d*. (**C**) Schematics showing the different states of the pneumatically adhesive membrane, which controls the adhesion from an OFF to ON state. (**D**) Underwater adhesion results from an octopus-inspired adhesive, which shows an adhesion switching ratio of 450**×** from the ON to OFF state. Error bars represent the SD for *n* = 3.

Here, we introduce an octopus-inspired underwater adhesive system composed of switchable adhesive elements coupled with a sensory system, processing, and control for autonomous adhesive activation and release (Fig. 1A). The adhesive element consists of a compliant, silicone stalk capped with a soft, pneumatically actuated membrane to control adhesion. These adhesive elements are tightly integrated with an array of micro–light detection and ranging (LIDAR) optical proximity sensors and a microcontrol for real-time object detection and control of adhesion. This tightly integrated system mimics the nervous system, enabling the system to intelligently control multiple adhesive elements to achieve dexterous manipulation in dry and wet environments. When an object is sensed at a programmed distance (d*), the adhesive membrane is triggered. This enables autonomous activation of adhesion through a prescribed control loop for rapid attachment and controlled release by tuning the state of the membrane (Fig. 1B). By applying positive pressure, we inflate the membrane for negligible adhesion; alternatively, we apply negative pressure to increase the volume of the adhesive element at the interface, creating a suction pressure and enhancing adhesion (Fig. 1C). This octopus-inspired mechanism enables adhesive stresses greater than 60 kPa underwater, with an adhesive switching ratio over 450× from the ON to OFF state (Fig. 1D). Reversibility is demonstrated over multiple cycles and rapid switching times <50 ms are achieved from a fully ON state to a released/OFF state. By tuning sucker compliance through stalk architecture, reliable attachment to off-angle substrates is enabled with reduced preloads with independent control of adhesive strength and work of separation providing reliable adhesion under nonideal conditions. The tight integration of sensors, processing, and control with rapidly switchable adhesives creates new opportunities for dexterous manipulation of underwater objects in compact systems without prior knowledge of the environment. This functionality is demonstrated in a wearable adhesive glove where we show the ability to pick up and release a variety of items underwater including flat, curved, rigid, and soft objects. These capabilities mimic the advanced manipulation, sensing, and control of cephalopods and provide a platform for synthetic underwater adhesive skins that can reliably manipulate diverse underwater objects.

## RESULTS

### Switchable adhesive fabrication and characterization

**Fig. 2.**
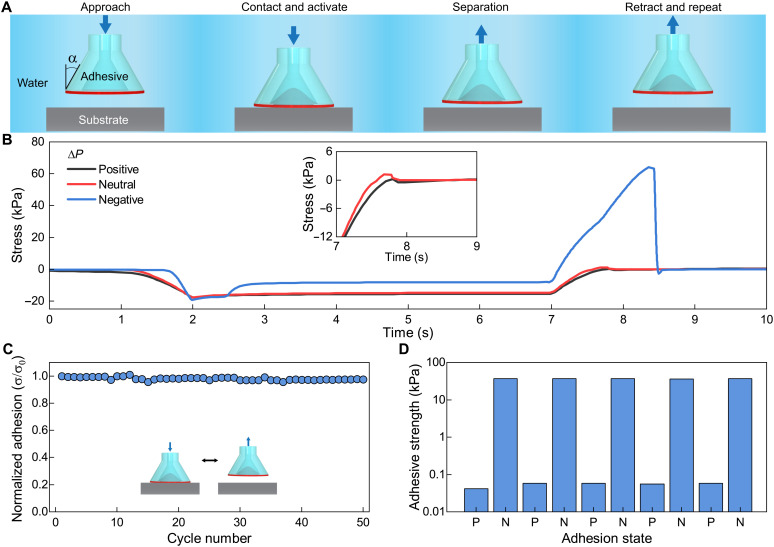
Characterization of switchable underwater adhesive. (**A**) Schematic showing the test procedure for underwater adhesive characterization. (**B**) Stress versus time plot displaying the preload, dwell, and retraction data for three different pressurized states. (**C**) Adhesion cyclic test of an α = 15° adhesive element for the negative state showing reusability over 50 cycles without degradation. (**D**) Cyclic adhesion test of an α ***=*** 15° adhesive alternating between positive and negative membrane states for high strength and release.

Adhesive elements were made from silicone elastomers, with the stalk being created with Dow Corning Sylgard 184 elastomer and the membrane from a more deformable Smooth-On Dragon Skin elastomer to accommodate large deflections. The stalk was fabricated by three-dimensional (3D) printing molds with prescribed geometry and then casting and curing the silicone elastomer. The stalk angle α is defined as the angle of the stalk near the contact surface (see Fig. 2A). The membrane was cast, partially cured, and then bonded to the stalk (further details in Materials and Methods). The adhesive element was then connected to a pressure source that supplies positive, neutral, and negative pressure to control the shape of the active membrane.

Adhesion strength of the adhesive elements was characterized for positive, neutral, and negative membrane pressurized states. This was performed on a custom testing setup that fully submerged the adhesive and substrate and pneumatically controlled the membrane state. The schematic of Fig. 2A illustrates the sequence of testing for an adhesive element with negative pressure (ON state). First, the adhesive approached an acrylic substrate until a predefined preload was reached. Next, a negative pneumatic pressure was applied to activate the adhesive. The adhesive was then held in place for 5 s and subsequently pulled from the substrate until separation (movie S1). The results for a positive, neutral, and negative pressure on an adhesive element with a stalk angle of α = 15° are shown in Fig. 2B. Here, we see that the negative state results in an adhesive stress above 60 kPa. This is in contrast to the low adhesive stresses for the positive and neutral states. The plot inset in Fig. 2B shows a zoomed-in view of the pull-off region, indicating that the positive state produces a lower adhesive strength than the neutral state due to the inflation and reduced contact with the substrate. This mechanism functions under both dry and underwater conditions (fig. S1). As we are using soft elastomer materials for the membrane and stalk, the adhesion is reversible and durable. To demonstrate the reversibility of the adhesives, we performed a cyclic experiment where negative pressure was applied and adhesive strength was measured over 50 consecutive experiments. We select an α = 15° and see in Fig. 2C that the adhesive was consistent over the tested cycles. The adhesive strength was normalized by the first cycle and we did not observe any degradation. By controlling adhesion through the active membrane, we can rapidly switch between high and low adhesive states. We programmed the pneumatic system to first perform an experiment with positive pressure and then switch to a negative pressure for the next cycle. This results in the ability to actively switch between a low and high adhesion state repeatedly over five cycles for each state as shown in Fig. 2D. Together, these results show the ability to generate substantial adhesive strength underwater, to be reusable over many cycles, and to achieve reversible switching between high and low adhesive states.

### Switchable adhesion strength, toughness, and release

**Fig. 3.**
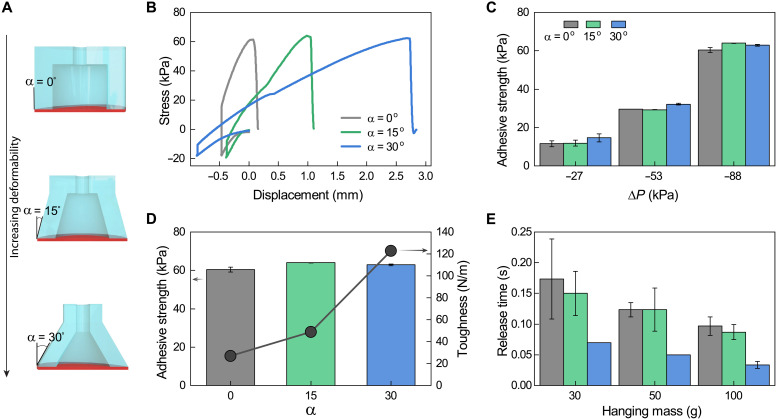
Underwater adhesion strength, toughness, and release. (**A**) Schematic showing the adhesive element’s change of stalk angle from 0° to 30°. (**B**) Graph representing the displacement versus adhesive force for each of the three stalk angles. (**C**) Adhesive strength for all three stalk angles under various negative pressures. (**D**) Plot showing the ability to achieve high adhesive strength and tunable adhesion toughness by changing the stalk angle α (data are for Δ*P* = −88 kPa). (**E**) Release times for three different masses for all three stalk angles. Error bars represent the SD for *n* = 3.

In unstructured environments, it is important that the adhesives are tolerant to angular misalignment. One way to improve contact creation is by increasing adhesive deformability. Here, we tune the compliance of the adhesive elements through stalk shape variation by changing the stalk angle (α). Figure 3A shows adhesive elements where α varies between 0°, 15°, and 30° while maintaining a constant contact area and height. We first evaluate the effect of stalk angle α on the ON state adhesive stress on a flat surface using an equivalent preload. Figure 3B shows underwater contact adhesion experiments where changing α adjusts the compliance of the adhesive element both as it is compressed and retracted. We find that relative to the stiffness of the α = 0° stalk, the stiffness during retraction decreases by a factor of 2.2× and 5.4× for α = 15° and 30°, respectively. We further simulate the deformation of adhesive elements during retraction in finite element (FE) analysis. These results show a decreasing stiffness with increasing stalk angle, where the stiffness decreases 5.1× as stalk angle changes from 0° to 30°, showing excellent agreement with the experimental results across this range (fig. S2C). Both experiments and simulation show the ability to control contact compliance by changing the stalk angle.

The influence of α and pneumatic pressure (Δ*P*) on adhesive strength is shown in Fig. 3C. First, we find that for a given α, increasing the magnitude of the negative pressure results in greater adhesive strengths. This enables controllable adhesion strength by tuning the applied negative pressure. Pull-off velocity can also tune adhesive strength, where we find increasing adhesive strength with increasing pull-off velocity (fig. S3). Second, we find that for a given Δ*P*, the adhesive strength remains the same irrespective of α. Even for the maximum negative pressure, a similar adhesive stress of greater than 60 kPa is found for all three stalk angles. We went as low as 88 kPa in this study as it balanced the time to pump down, considerations for sealing, and negative pressure generation.

Although all the samples have the same maximum adhesion strength, the compliant adhesive element with α = 30° is advantageous as it provides for a tougher adhesive and the ability to conform to different surfaces with angular misalignment. The toughness is evaluated by the overall work needed to remove the adhesive during separation (fig. S4). The most compliant sample of α = 30° takes more work for removal even as the adhesive stress is the same as other angles (Fig. 3D, where the black line with circular symbols refers to toughness). This effective change in stiffness allows for the α = 30° adhesive element to exhibit a 4.6× higher work of fracture relative to the α = 0° sample under the same loading conditions. This shows the capability to increase the work of fracture of the adhesive by changing the sample geometry without sacrificing adhesive strength.

The adhesives can also be switched from an ON state to an OFF state while supporting a load. Rapid switching performance for different stalk angles α = 0°, 15°, and 30° was examined using masses of 30, 50, and 100 g. We performed the underwater switching tests by lifting a mass in the negative pressure state and then switching to the positive pressure state. For this experiment, the positive pressure was approximately 5 kPa and the negative pressure was −88 kPa. This transition inflates the membrane to reduce the adhesion and rapidly drops the mass. The time required to drop a mass after triggering the adhesion change is compiled in Fig. 3E. Here, we see that release time decreases for increasing mass, and that the α = 30° shows the most rapid release (movie S2). All stalk angles release the mass in less than 200 ms, with the α = 30° adhesive releasing the 100-g mass in less than 40 ms, showing the ability to rapidly switch from a high to low adhesion state underwater.

Together, these results show the ability to achieve high adhesion strength and toughness while also being able to rapidly (<0.1 s) and controllably switch adhesion to the OFF state. This combination of strength, toughness, and release is often contradictory in adhesives, yet is achieved here through the combination of stalk geometry and active control of membrane curvature in these soft, octopus-inspired adhesives. This represents an exceptional combination of underwater adhesion switching characteristics, which is uniquely enabled by the ability to control deformation through the stalk geometry for toughness, while being able to actively control the membrane geometry for strength and rapid release.

### Switchable adhesion under nonideal conditions

**Fig. 4.**
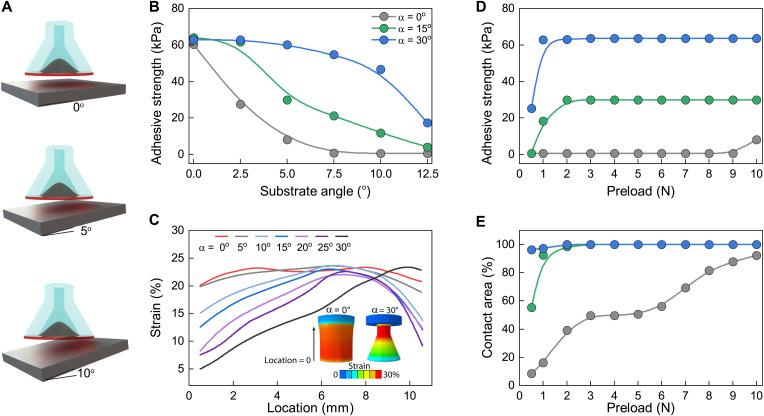
Adhesion in nonideal conditions. (**A**) Schematic showing the change of substrate angle from 0° to 10°. (**B**) Representation of the adhesive strength as the substrate angle changes to 12.5° for all three stalk angles. (**C**) Graph showing the strain percentage as a function of the location of the adhesive element under adhesion. The insets show the strain distribution on the FE analysis models for α = 0° and α = 30°. (**D**) Graph showing the adhesion strength dependence on preload evaluated on a substrate angle of 5°. (**E**) Percentage of contact area on the 5° substrate as a function of preload for all three stalk angles.

In unstructured environments, adhesives may not always be well aligned with substrates of interest. We study the misalignment tolerance of the adhesives by characterizing the adhesion properties against inclined substrates with different angles relative to the plane of the adhesive element (Fig. 4A). Figure 4B presents the adhesive strength as a function of substrate angle ranging between 0° and 12.5° for the same preload of 3 N (17 kPa). The adhesive strength of the α = 30° stalk is maintained for inclined angles up to 5° and is the only element capable of adhering with a substrate inclination >10°. The α = 0° adhesive fails to achieve any adhesion above inclined angles of 5°, as the element is no longer able to create contact, highlighting the importance of compliance for contact generation. The FE model is further used to characterize the deformation along the stalk for different stalk angles (α). Figure 4C shows the strain profiles along the stalk, where the location axis starts from the tip of the membrane (see fig. S2 for more details). The inset of Fig. 4C shows the deformed adhesive element for α = 30°. An equal displacement of 2.5 mm is used for all angles to compare strain profiles. The smaller angles (e.g., 0°) show relatively uniform strain distribution with high strain near the contact area (near the 0-mm position). Conversely, for larger stalk angle α (e.g., 30°), the strain is noticeably smaller near the contact location, which gradually increases toward the base. This behavior indicates that adhesives with larger α experience smaller strain near the contact zone, while the thin region of the stalk elongates substantially. The stress distribution in fig. S2 also shows lower stress near the contact zone and greater stress in the thin stalk region for α = 30°. These results indicate that the negligible strain of the α = 30° adhesive element near the contact area ensures minimal disturbance of the flexible membrane for robust adhesion performance, even when the element is substantially misaligned.

We next used a constant substrate angle of 5° and examined the adhesive strength dependence on preload. Here, the amount of preload was increased from 0.5 to 10 N and we report the maximum adhesive strength for each stalk angle. Figure 4D shows that the α = 30° element achieved adhesion even for a low preload of 0.5 N (2.8 kPa) and then reached a maximum adhesion strength above 60 kPa for 1-N (5.7-kPa) preload. For the α = 15° element, adhesive strength begins to develop for the 1-N preload and then plateaued at a moderate adhesive strength of 30 kPa at a 2-N preload. The 0° adhesive element requires at least 10-N preload to develop any adhesion strength and even at that point only shows a low adhesion strength of 10 kPa, substantially smaller than the more compliant elements. The preload on adhesive elements is an important factor for creating contact with the substrate. This was further examined using FE analysis to determine contact area as a function of preload. Here, we calculated the contact area as a ratio of contact nodes to total nodes of membrane during compression of adhesive elements onto a 5° inclined surface (more details in fig. S5).

The summarized contact area analysis for α = 0°, 15°, and 30° is presented in Fig. 4E. By comparing the adhesive strength and contact area in Fig. 4E, we find that successful adhesion develops when 90% of the adhesive is in contact with the substrate. We interpret this condition as the minimum contact area required to ensure the membrane component is in contact with the substrate. The stiffer α = 0° and 15° elements require high preload to satisfy the contact area condition to initiate adhesion with inclined substrates. In contrast, the highly flexible adhesive elements having α = 30° can meet the contact area requirement with low preload. This reduced preload is considerably advantageous as it allows for robust contact and strong underwater adhesion without pressing hard into substrates. These results are clear indicators of the conformal adhesion mechanism of the octopus-inspired adhesive elements and point to the importance of stalk design to strongly adhere underwater in unstructured environments.

### Adhesive skin for underwater gripping and manipulation

**Fig. 5.**
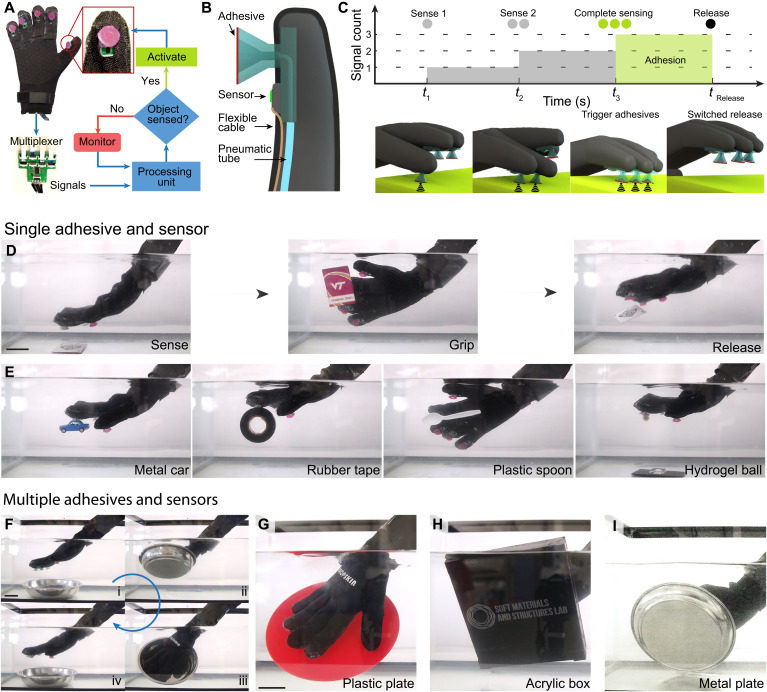
Octopus-inspired adhesive skin for intelligent underwater manipulation. (**A**) Wearable adhesive glove with integrated adhesives, sensors, processing, and control showing the logic layout to activate adhesion. (**B**) Cross section of a finger illustrating the embedded sensor and adhesive element. (**C**) Sequence of the sensorized adhesive showing the adhesion triggering after complete sensing by three sensors followed by switched release. (**D**) Single adhesive activation mode to sense, grip, and release a lightweight paper card in an underwater environment. (**E**) Underwater manipulation with a single adhesive and sensor to adhere and pick up a metal car, rubber tape, plastic spoon, and a hydrogel ball. (**F**) Demonstration of multiple adhesives and sensors on the adhesive glove to grip, lift, and release a large metal bowl in water. (**G**) Underwater manipulation using the full adhesive glove for a plastic plate, (**H**) an acrylic box with a laboratory logo, and (**I**) a metal plate. Scale bars, 5 cm.

The octopus-inspired adhesive elements were then tightly integrated with a sensorized skin to create a wearable glove for autonomous adhesion control and dexterous manipulation of underwater objects as shown in Fig. 5A. Each finger of the glove consists of an active adhesive element and micro-LIDAR optical sensor for proximity detection. The array of optical proximity sensors were connected to a microcontroller using a multiplexer where the proximity data were collected to determine whether an object has been detected. If an object was within the sensing range, a digital signal was sent to activate a solenoid-controlled pneumatic device for rapid activation of the adhesive elements. A cross section of a finger from the sensorized glove is shown in Fig. 5B. The optical proximity sensor was fixed to the elastomeric platform that contained the adhesive element. The flexible sensor cable and pneumatic tube were routed inside the glove. Adhesion with complex geometry was aided by the ability of α = 30° adhesive elements to conform to a surface with a small preload. Sensorized gripping with the glove is illustrated in Fig. 5C by a sequence of schematics and corresponding time plot. Different adhesive activation modes can be achieved by controlling the proximity range for object detection and actuation timing for a selected group of sensors. For instance, we program the adhesive elements to activate after three sensors detect an object as shown in Fig. 5C. Notice that the adhesives are inactive in the first three sensing instances (*t* < *t*_3_). When three sensors recognize a substrate at *t*_3_, a digital signal is sent to actuate the pneumatic trigger, which initiates rapid adhesion. The release can also be performed by switching off the adhesion at *t*_Release_.

Both the sensorized skin and adhesive elements function while submerged, enabling the wearable glove to manipulate diverse objects underwater. To manipulate delicate and lightweight objects, we use a single sensor mode to activate the adhesives. Figure 5D shows that the index finger can recognize a small card and trigger adhesion. The negatively pressurized adhesive attaches to the card and then the user rotates their hand to show the “VT” logo (middle image of Fig. 5D). The card is then released on demand (Fig. 5D). Figure 5E shows underwater manipulation of other small and lightweight items with different shapes and materials. These items include a metal toy car, cylindrical rubber tape, the doubly curved convex portion of a plastic spoon, and an ultrasoft hydrogel ball, demonstrating adhesion to flat, cylindrical, convex, and spherical surfaces across hard and soft materials (movie S3).

It is also possible to grip larger objects with all of the adhesive elements by reconfiguring the sensor network to use all sensors for object detection. Here, we program the microcontroller to actuate the pneumatic trigger after three of the adhesives sense the proximity of an object. This mode ensures contact of all the adhesive elements with the substrate before activating adhesion. Figure 5F shows the use of a fully functional adhesive glove for gripping the concave surface of a metal bowl. The adhesives approach the object [Fig. 5F (i)] and then autonomously activate adhesion to enable easy lifting and handling of the bowl [Fig. 5F (ii and iii)] before activating release [Fig. 5F (iv)]. This functionality is repeated in Fig. 5 (G to I) to manipulate a plastic plate, acrylic box, and metal plate, demonstrating dexterous underwater manipulation of different materials with a range of surface reflectivity (see movie S4).

## DISCUSSION

We have introduced an octopus-inspired underwater manipulation system by tightly integrating sensing, processing, and control with rapidly switchable adhesives. This is enabled by adhesives that switch adhesion 450× from the ON to OFF state quickly (<0.1 s) with the ability to be reused over multiple cycles. By tuning sucker compliance through stalk architecture, we achieve reliable attachment in unstructured environments at low preloads. This functionality was demonstrated in a wearable adhesive glove to autonomously activate adhesion to pick and release a variety of items underwater including flat, curved, rigid, and soft objects. These capabilities mimic the advanced manipulation, sensing, and control of cephalopods and provide a platform for synthetic underwater adhesive skins that are able to manipulate diverse underwater objects.

One of the enabling features of this octopus-inspired adhesive system is real-time object detection coupled with rapidly switchable adhesives. This allowed for manipulation of diverse objects at time scales relevant to human movement. This was achieved because of the low preload required to activate adhesion on different substrates by optimizing the architecture of the adhesive stalk. This low preload adhesive activation coupled with the object detection through sensing and rapidly switchable adhesives is an important combination to achieve underwater manipulation with autonomous gripping and release.

Tuning stalk compliance also enabled independent control of adhesive strength and toughness. This increase in toughness was achieved while maintaining the ability to rapidly release objects. This combination is unusual as higher adhesion toughness is typically achieved with enhanced inelastic dissipation, which can make release difficult and typically increases switching time. Therefore, control over adhesive strength, toughness, and release is critical for efficient manipulation. The strength allows for relatively heavier objects to be manipulated, the toughness allows for tolerance to perturbations during manipulation where the adhesive can deform while still grasping the object, and the ability to trigger a low adhesive state allows for objects to be released despite the higher strength and toughness. This combination of controlling strength and toughness with rapid release is an exceptional combination of adhesive properties, yet is achieved in this system and is extremely advantageous for underwater manipulation. Systematic evaluations of negative/positive pneumatic pressure differential, membrane geometry, stalk geometry, water depth, and object characteristics would be an excellent area for future work to further establish the full range of ON/OFF ratio characteristics for the switchable adhesives. Furthermore, exploration of microfabrication strategies could enable device downscaling and integration with microfluidic channels could allow for multiplexing the pneumatic system.

Although this study is focused on optical sensors, different sensing modalities could also be used in the future. Chemical or mechanical sensors could be synergistic, and this could be particularly interesting as it is known that the octopus displays a diverse set of vision, chemical, and mechanical sensing during manipulation. There are also future opportunities to incorporate haptic feedback into this system to alert a user when adhesives are activated, and this will allow for tuning of the control scheme for customizable underwater manipulation. Further, the use of pneumatic activation for the adhesives was the focus of this current work. We anticipate that other types of switchable adhesives could be used, as long as the switching time (i.e., the time to activate or release the adhesive) is on the order of seconds or less, which allows for active manipulation without needing to prime the system or wait extended amounts of time in contact. Also, we anticipate that future embodiments of the octopus-inspired adhesive skin could be deployed as an unthetered system. Recent examples of untethered soft material actuation have shown pneumatic systems (pumps, valves, electronics, and batteries) on the order of 500 g that can be carried in a small bag or within the soft robot itself (*50*, *51*). Miniature pumps can run on 10 W and recent work has shown low-power soft pumps that consume 100 mW (*52*). Finally, the pneumatic membrane in our adhesives provides a closed system, which could allow for the pneumatic system to be powered off after activation (i.e., no power consumed during gripping/manipulation), which could provide power savings. These future directions could allow for additional functionalities for robotic manipulation, manufacturing, and health care for programmed or autonomous manipulation of surfaces, materials, and tissues in dry or wet environments.

## MATERIALS AND METHODS

### Adhesive element manufacturing and preparation

Molds were created from a DLP 3D printer (B9 Creations) with variations in the stalk angles α: 0°, 15°, and 30° with a sucker diameter of 15 mm. The adhesive elements were fabricated with polydimethlysiloxane (PDMS) (Sylgard 184 with a 10:1 ratio), by pouring elastomer into 3D-printed molds and curing at 80°C for 8 hours. The PDMS was removed and treated with oxygen plasma for 1 minute before placement onto a 500-μm silicone membrane (Dragon Skin 00-30, Smooth-On), which was partially cured at 80°C for 2 min. The adhesive element and partially cured membrane were cured at 80°C for 4 hours. A 20-gauge needle attached with pneumatic tubing was inserted into the base of each sample where an air channel was located. A silicone adhesive (Sil-Poxy, Smooth-On) was used to seal the inserted needle to the sample.

### Adhesive testing

Adhesive elements were tested through normal adhesion experiments on an Instron 5944 load frame. Adhesive elements were lowered onto an acrylic substrate and compressed to a force of 3 N or ∼17 kPa and held for 5 s while the desired pneumatic state was activated. The sample was then retracted at 1 mm s^−1^ until separation. Each sample was tested with positive pressure, neutral pressure, and 27, 53, and 88 kPa of negative pressure. An additional test was conducted outside of water with the same setup to determine the effects of a dry substrate. Angled substrate tests were performed with a tilted, acrylic substrate at 2.5°, 5°, 7.5°, 10°, and 12.5°. Each sample was lowered onto the substrate with a preload of 3 N and subjected to the maximum negative pressure of 88 kPa.

### FE analysis

The computational models of adhesive elements were developed using the FE program Abaqus/Standard (Simulia, Providence, RI) as 3D deformable bodies. We use eight-node linear brick elements C3D8R, which uses reduced integration with enhanced hourglass control. Figure S2A shows an adhesive element for stalk angle α = 30° where the arrow represents the direction for stress and strain profiles. The stalk (PDMS) and membrane (Dragon Skin) of the adhesive element were formulated using hyperelastic Yeoh model (*53*) materials. The material coefficients *C*_10_ = 0.19 MPa, *C*_20_ = 0.21 MPa, and *C*_30_ = 0.01 were used for PDMS and *C*_10_ = 0.37 MPa, *C*_20_ = 0.005 MPa, and *C*_30_ = 0 were used for Dragon Skin. The semi-rigid adhesion contact was replicated using spring boundary condition (1 N/mm for each spring) at the bottom surface of the membrane. This value was tuned to fit with the experimental result shown in fig. S2B.

The contact area analysis in FE was performed using the same material properties for the adhesive element. However, the membrane boundary conditions were changed to a friction contact between the adhesive surface and the inclined substrate. A nonlinear friction coefficient was used as a function of preload (see fig. S5A), which was used for the analysis of all stalk angles.

### Wearable adhesive glove

The wearable adhesive glove is developed from a neoprene wetsuit glove (3-mm NeopSkin Water Gloves), which hosts the adhesive elements and sensors in each finger. The adhesive elements were cut into rectangular pieces to fit the glove fingers and flexible pneumatic tubes with 0.8 mm inside diameter were inserted at the base of the adhesives. The sensing in the glove is achieved by using a micro-LIDAR optical sensor (STMicroelectronics, VL6180X) that is wired together using flat flexible cables (Molex). A sensor is attached to each adhesive element (see Fig. 5A) using silicone adhesive (Smooth-On Sil-Poxy) with an unobstructed field of view. The sensors and flat flexible cable joints were spray-coated with a thin, waterproof layer of conformal coating (Humiseal 1A33 Aerosol). The pneumatic tubes from the adhesives were combined using a heat shrink wrap and fit to a solenoid valve (Spartan Scientific 2-Way/2-Position Valve) through a plastic tube (PureSec CCK RO Tubing). The inlet of the solenoid valve is attached to a vacuum pump. Multiple optical sensors, which have a fixed I2C address, were connected to a single microcontroller (Microchip, ATmega2560) using a bidirectional multiplexer (Texas Instruments TCA9548). The microcontroller is used to control the solenoid operated pneumatic system based on the optical sensor network feedback.

### Statistical analysis

The meaning of all error bars and how they were calculated are described within the captions of the figures in which they occur.
