# Supplementary material for: Octopus-inspired adhesive skins for intelligent and rapidly switchable underwater adhesion
Source: Sci Adv. 2022 Jul 13;8(28):eabq1905. doi: 10.1126/sciadv.abq1905 (PMC9278861; doi:10.1126/sciadv.abq1905)
Supplement: Supplementary file 1 — Figs. S1 to S5 [file sciadv.abq1905_sm.pdf]

Science Advances
AAAS

Supplementary Materials for

Octopus-inspired adhesive skins for intelligent and rapidly switchable underwater adhesion

Sean T. Frey *et al.*

Corresponding author: Michael D. Bartlett, mbartlett@vt.edu

Sci. Adv. **8**, eabq1905 (2022)
DOI: 10.1126/sciadv.abq1905

The PDF file includes:

Figs. S1 to S5
Legends for movies S1 to S4

Other Supplementary Material for this manuscript includes the following:

Movies S1 to S4

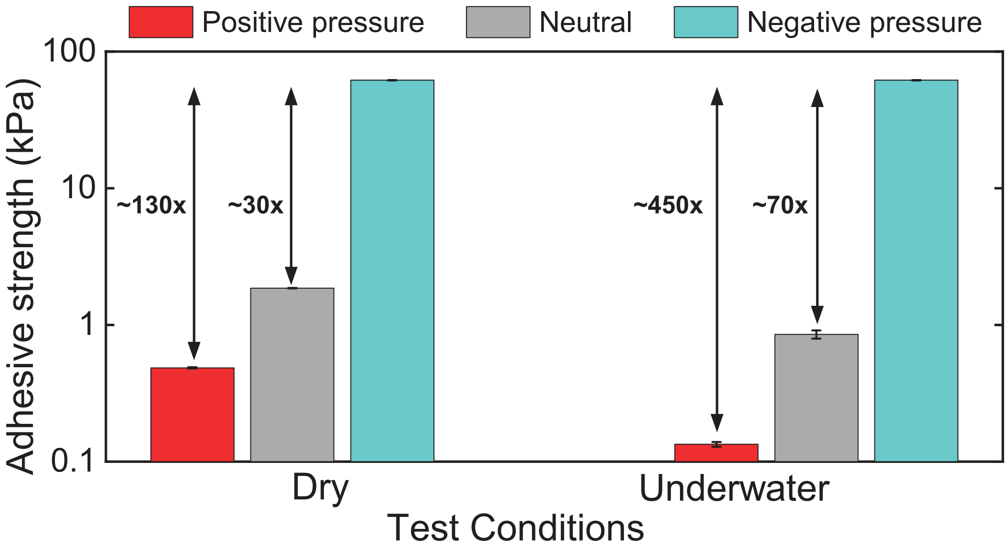

Fig. S1 Adhesive strength in dry and underwater conditions. Adhesive strength for dry and underwater conditions for positive, neutral, and negative pneumatic pressure.

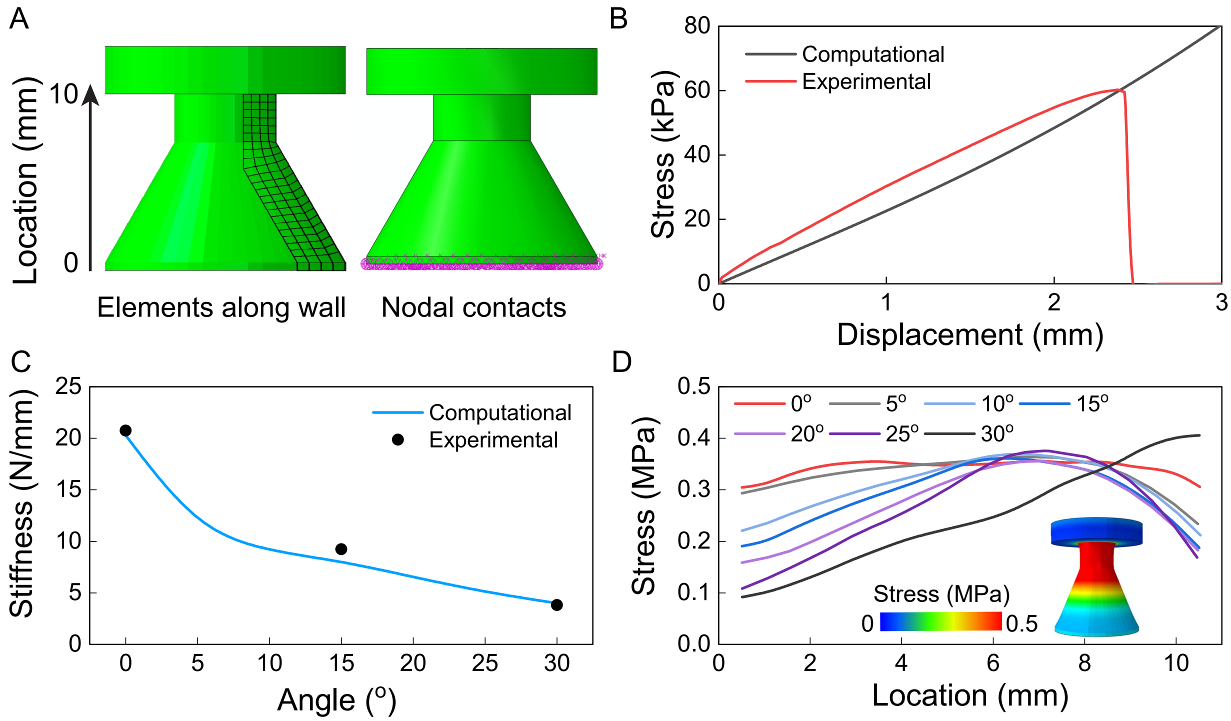

Fig. S2 Computational analysis for effect on stalk angle on the mechanical behavior of adhesive elements. (A) Model of an adhesive for $\alpha = 30°$ where the left schematic presents a strip of elements along the arrow (red borders). The right schematic shows the spring support by the nodal contacts at the membrane. (B) Comparison of stress-displacement relation provides the validation of the computational model. (C) Relation of adhesive stiffness as a function of stalk angle. (D) Stress profiles for the elements along the stalk (arrow indicating location) for different stalk angles.

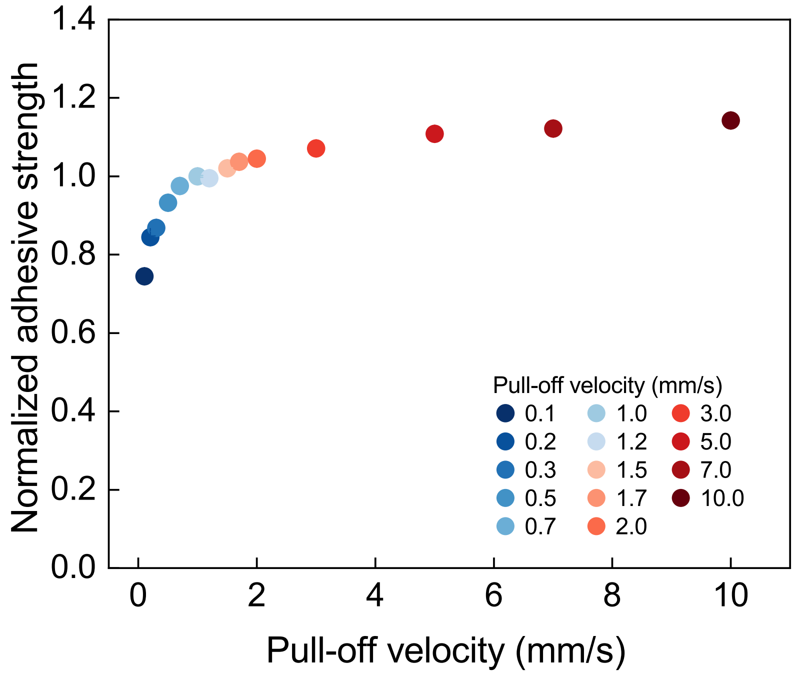

Fig. S3 Dependence of underwater adhesive strength on pull-off velocity. Normalized adhesive strength for a range of pull-off velocities from 0.1 to 10 mm/s (stalk angle $\alpha = 30°$). Data is normalized by the adhesive strength at a velocity of 1 mm/s, which was the standard testing rate for underwater adhesion experiments.

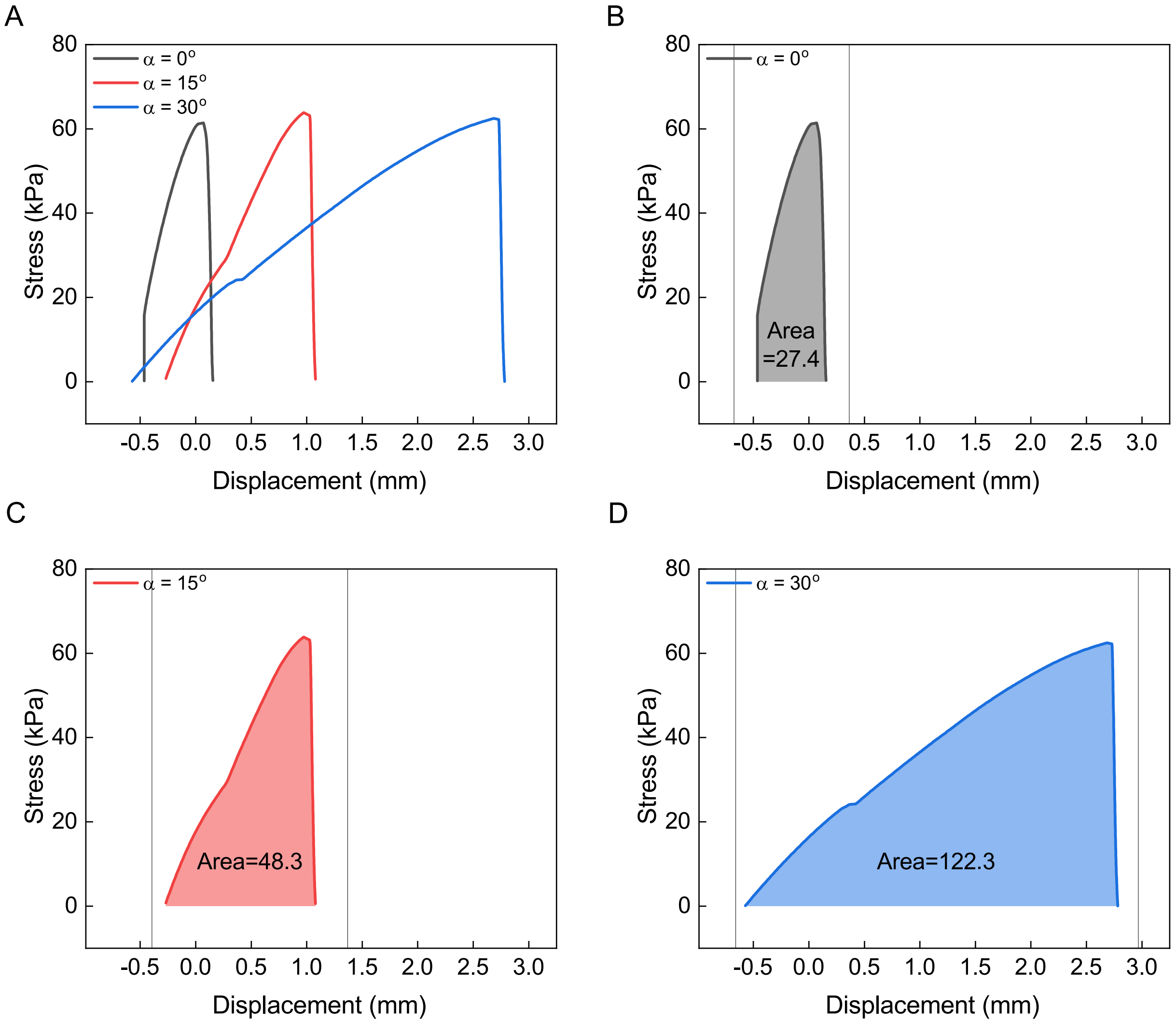

Fig. S4 Analysis of toughness for suckers with different stalk angles. (A) Stress vs displacement curves for stalk angles $\alpha = 0°$, $15°$, $30°$ (B) Area under curve calculated to determine toughness by using curve integration in OriginPro software for $\alpha = 0°$ (C) $\alpha = 15°$ (D) $\alpha = 30°$.

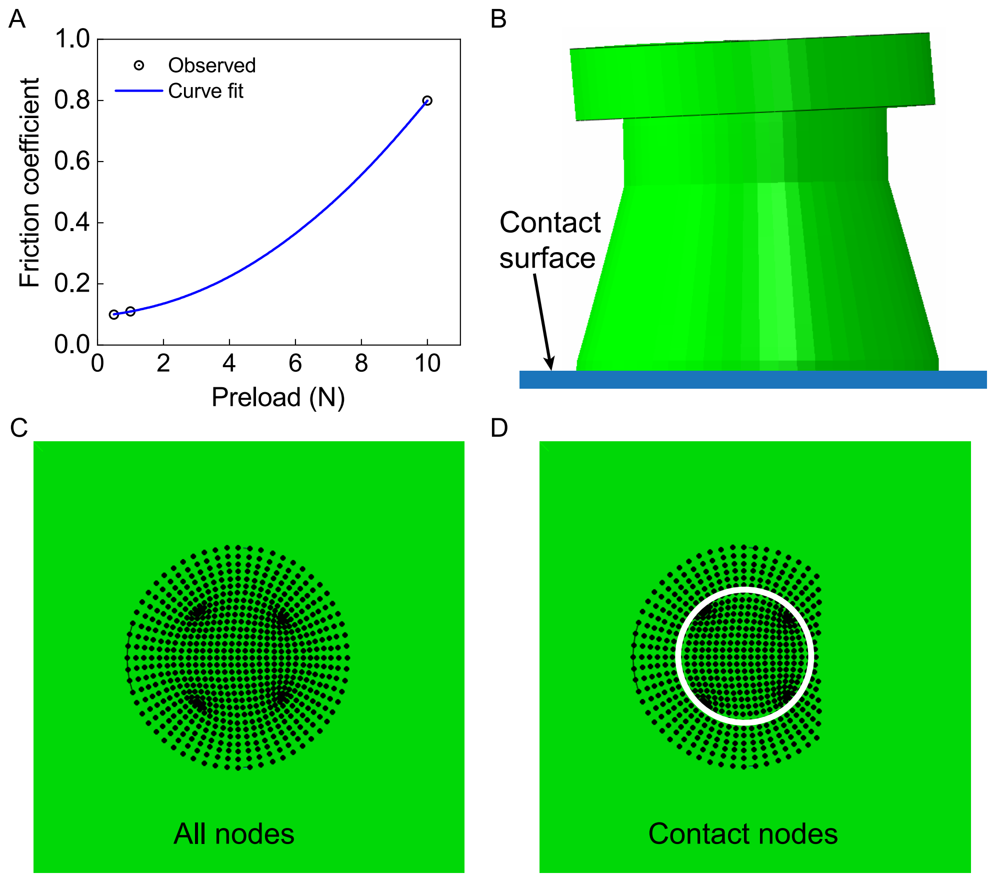

Fig. S5 Analysis for effect of preload on contact area. (A) Calibration of friction coefficient to use in the FE model where the experimentally observed data is used to fit the friction coefficient. (B) A 15° adhesive element model is compressed at 5° inclination on a surface (blue plate) (C) A set of all the nodes (red dots) at the surface of membrane. (D) The group of membrane nodes that make contact with contact surface. The white circle represents the inner border of the adhesive stalk.

Movie S1: Underwater adhesion test. Side profile view of an underwater adhesion experiment with adhesive element stalk angle $\alpha = 30°$.

Movie S2: Hanging mass release time. Side profile view of an underwater adhesion experiment where a 30 g, 50 g, and 100 g mass are picked up and released (stalk angle $\alpha = 30°$). The red LED in the foreground indicates activation (negative pressure) while the green LED indicates release (positive pressure).

Movie S3: Octopus-inspired adhesive glove with single-sensor activated adhesion. Side profile view of a single adhesive and sensor activation mode demonstration to sense, grip, and release objects in an underwater environment.

Movie S4: Octopus-inspired adhesive glove with multi-sensor activated adhesion. Side profile view of a multi-sensor adhesive and sensor activation mode demonstration to sense, grip, and release objects in an underwater environment.
